# Genomic Characterization of Antimicrobial Resistance in Food Chain and Livestock-Associated *Salmonella* Species

**DOI:** 10.3390/ani11030872

**Published:** 2021-03-18

**Authors:** Thobeka P. Mthembu, Oliver T. Zishiri, Mohamed E. El Zowalaty

**Affiliations:** 1 Discipline of Genetics, School of Life Sciences, College of Agriculture, Engineering and Sciences, University of KwaZulu-Natal, Private Bag X54001, Durban 4000, South Africa; thobeka22m@gmail.com (T.P.M.); Zishiri@ukzn.ac.za (O.T.Z.); 2 Department of Clinical Sciences, College of Medicine, University of Sharjah, Sharjah 27272, United Arab Emirates; 3 Zoonosis Science Center, Department of Medical Biochemistry and Microbiology, Uppsala University, Uppsala SE 751 23, Sweden

**Keywords:** antimicrobial resistance, *Enterobacteriacae*, *Salmonella*, foodborne, food chain, livestock zoonosis, *mcr-1*, carbapenem, colistin, food animals, genotyping, plasmid, humans, resistance, One Health

## Abstract

**Simple Summary:**

In the era of the COVID-19 global pandemic, antimicrobial resistance is looming as an imminent threat and challenge to human public health. Antimicrobial resistance is a major global threat of increasing concern to human and animal health. It also has implications for both food safety and food security and the economic well-being of millions of humans. Among other zoonotic infectious diseases, antimicrobial resistance in food chain and livestock-associated pathogens such as *Salmonella* is of great concern. In the present study, the genomic characterization of antimicrobial resistance in food chain and livestock-associated *Salmonella* was summarized. Several antimicrobial resistance determinants were reported in *Salmonella* isolated from food chain animals and livestock. Monitoring of antimicrobial resistance in *Salmonella* in livestock and food chain animals through genomic characterization is significant to control and protect humans from the threat of antimicrobial resistance. *Salmonella*, a foodborne zoonotic enterobacterium species can transmit antimicrobial resistance from the microbiome of animals to humans. This study summarizes the genomic characterization of antimicrobial resistance in *Salmonella* species with special focus on resistance against carbapenems and colistin which are the last resort antibiotics used against infections caused by multidrug resistant bacteria. The present review aims to draw attention to prudent use of antibiotics, a good example of the One Health concept.

**Abstract:**

The rising trend of antimicrobial resistance (AMR) by foodborne bacteria is a public health concern as these pathogens are easily transmitted to humans through the food chain. Non-typhoid *Salmonella* spp. is one of the leading foodborne pathogens which infect humans worldwide and is associated with food and livestock. Due to the lack of discovery of new antibiotics and the pressure exerted by antimicrobial resistance in the pharmaceutical industry, this review aimed to address the issue of antibiotic use in livestock which leads to AMR in *Salmonella*. Much attention was given to resistance to carbapenems and colistin which are the last-line antibiotics used in cases of multi drug resistant bacterial infections. In the present review, we highlighted data published on antimicrobial resistant *Salmonella* species and serovars associated with livestock and food chain animals. The importance of genomic characterization of carbapenem and colistin resistant *Salmonella* in determining the relationship between human clinical isolates and food animal isolates was also discussed in this review. Plasmids, transposons, and insertion sequence elements mediate dissemination of not only AMR genes but also genes for resistance to heavy metals and disinfectants, thus limiting the therapeutic options for treatment and control of *Salmonella*. Genes for resistance to colistin (*mcr-1* to *mcr-9*) and carbapenem (*blaVIM-1*, *blaDNM-1*, and *blaNDM-5*) have been detected from poultry, pig, and human *Salmonella* isolates, indicating food animal-associated AMR which is a threat to human public health. Genotyping, plasmid characterization, and phylogenetic analysis is important in understanding the epidemiology of livestock-related *Salmonella* so that measures of preventing foodborne threats to humans can be improved.

## 1. Introduction

Antimicrobial resistance continues to be a challenge to public health due to the ever-rising trends of multi-drug resistant bacterial isolates in clinical settings. Antimicrobial resistance is propagated by the improper administration of antibiotics in human medicine however, the agricultural sector, through animal husbandry, is the origin of antimicrobial resistance and the major contributor of antimicrobial resistance pathogens to humans. Overuse of antimicrobial agents in livestock led to the emergence of drug resistant bacteria which constitute part of the gut microbiota. Antibiotic administration should be based on susceptibility results of the causative bacteria. The food chain is the main route in which foodborne pathogens such as non-typhoidal *Salmonella* spp. are transmitted from animals to humans.

Colistin and carbapenems are critical antibiotics in human medicine which are used to treat multiple drug resistant (MDR) bacterial infections. Due to reported resistance to these antibiotics in the clinical settings, this study reviewed the reports of carbapenem and colistin resistant *Salmonella* in food animals with an aim of determining if there is a relationship between carbapenem and colistin resistant *Salmonella* isolated from humans and animals. Another aim of this review is to emphasize the role played by genomic characterization of livestock-associated and antimicrobial resistant pathogens in creating references for foodborne diseases outbreak tracing. Covered in this review are the genetic determinants of colistin and carbapenem resistance in *Salmonella* spp., the role played by the mobile genetic elements in mobilizing colistin and carbapenem resistance genes and the importance of genomic approaches in characterizing and monitoring the dissemination of antimicrobial resistance genetic determinants. 

## 2. Antibiotics Use in Livestock Production

The livestock is a significant source of protein for humans through the consumption of animal origin meat, eggs, and dairy products [1]. Monogastric animals and ruminants can convert the high fiber found in vegetation into protein [2]. Humans rely on livestock for meat and income generated from livestock by-products for the greater part of their livelihood.

The wellbeing of animals lies in the cautious practice of animal husbandry by the farmers. The well reared and healthy livestock produce meat, milk, and eggs that are nutritious to humans and reasonable income for the farmer [3]. Shelter, water availability, nutritious feed, disease prevention, and treatment measures must be considered by the farmers to ensure proper animal growth [3,4]. For disease treatment, antibiotics are supplied to animals in therapeutic doses for a short duration while for disease prevention, sub-therapeutic doses of antibiotics are added to water or feed for a longer duration in cases of suspected risk of infection [5]. Farmers also use antibiotics for growth promotion [6]. Prophylactic use of antibiotics is sometimes confused with growth promotion purposes, leading to a continuous supply of antibiotics to healthy animals [7]. As early as 2006, the European Union has banned the use of antibiotics for growth promotion. According to the WHO, healthy animals should only receive antibiotics to prevent disease if the disease has been diagnosed in other animals in the same flock or herd. Although antibiotic use has some benefits in livestock production, it also leads to the occurrence of antimicrobial-resistant bacteria. The practice of using antibiotics as growth promotion in agricultural should be prohibited and banned immediately to avoid exacerbation of the antimicrobial resistance challenge. 

## 3. Livestock Industry as a Source of Antimicrobial Resistance

The world faces the horrendous challenge of the emergence of multidrug resistant foodborne pathogens, which render antimicrobials ineffective in combating diseases [8,9]. Antimicrobial resistance is a public health concern as the world is slowly going back to the era before the discovery of penicillin [10]. Before Alexander Fleming discovered penicillin, even a minor injury was difficult to treat and would lead to death due to bacterial infection [10]. Injudicious use of antibiotics in agriculture and human medicine is the driving force behind antimicrobial resistance [6]. In livestock, antibiotics were registered for disease treatment, but they have been exploited for growth support and prophylaxis [10].

## 4. Outcomes of the Imprudent Use of Antibiotics in Livestock Production

The guts of eukaryotic organisms harbor many Gram-negative microorganisms that form the natural microflora, which aid in food degradation and digestion [11,12]. Some of the enteric bacteria are opportunistic pathogens and are induced by factors such as stress and antibiotics [13,14]. Due to short generation time, bacteria evolve faster than other organisms in the ecosystem [13,15]. Microorganisms have responded to evolution and acquired diverse mobilizable defensive and metabolic mechanisms against foreign attacks, including antibiotics [16]. Therefore, overuse of antibiotics in animal husbandry turns livestock into carriers of antimicrobial resistant enteric pathogens which become a problem for humans.

Rapid population growth results in the demand of meat, milk, and eggs, thus pressuring the livestock production industry [1,17]. The high demand tends to encourage swift and extensive farming using antibiotics [17]. According to Chattopadhyay, adding antibiotics to animal feed improves the feed conversion efficiency and growth rate of the animals, it also reduces the expenditure as the animals consume less feed than the animals that consume the feed that is not supplemented with antibiotics [6]. Some of the antimicrobials that are used for animal growth promotion and prophylaxis are closely related to therapeutics used in humans, which renders them ineffective due to resistance by bacteria [18]. One of the problematic antimicrobial-resistant pathogens is *Salmonella* spp. which causes gastroenteritis, enteric fever, and bacteremia [19].

## 5. Epidemiology of *Salmonella*

Salmonellosis is a burden to both developing and developed countries worldwide [20,21]. Global studies reported that Africa is leading with the prevalence of non-typhoidal *Salmonella* [21,22]. A global study by Ao et al. reported that 57% of invasive non-typhoidal *Salmonella* illnesses and deaths occurred in Africa in 2010 [20]. The high prevalence of lethal salmonellosis in Africa is associated with poor or primitive health care, human immunodeficiency virus, malnutrition, malaria, and antibiotic resistance [23]. Non-typhoidal *Salmonella* spp. has a broad host range and infect both humans and animals [24]. Animals are known to be the carriers of non-typhoidal *Salmonella* spp. as part of their natural microbiota [25]. Phylogenetic studies have revealed an evolutionary relationship between *Salmonella* spp. isolates from animals, the environment, and humans [26,27,28]. Djeffal et al. reported the clustering of 7 S. Heidelberg strains isolated from avian species and human isolates [26]. Eguale et al. reported the clustering of fluoroquinolone-resistant S. Kentucky strains isolated from poultry, cattle, and humans [27]. A study by Pornksukarom et al. unpacked the relatedness between the genomes of *Salmonella enterica* serovar Typhimurium, *Salmonella enterica* serovar Derby and *Salmonella enterica* serovar Schwarzengrund isolated from chicken, the environment, swine, and humans [28]. Another study by Ekwanzala et al. reported that in South Africa, farm settings share a significant number of antimicrobial resistance genes with clinical and environmental settings compared to clinical vs. environmental settings [29]. The previously cited studies concluded that animals are the reservoirs of *Salmonella* spp. and a source of human and environmental contamination. 

Global dissemination of *Salmonella* spp. is facilitated by travelers, imports, and exports of livestock and livestock products [30]. Some *Salmonella enterica* strains are restricted to a particular geographical area, such as invasive *Salmonella enterica* serovar Typhimurium Sequence Type (ST) 313 and the recently discovered African *Salmonella enterica* serovar Enteritidis strains [23,30]. Cases of *Salmonella enterica* serovar Typhimurium ST313 strains in the United Kingdom were however reported [30]. During an investigation of the African *Salmonella enterica* serovar Typhimurium ST313 isolated from humans, a dog, and an unspecified food source led to the conclusion that the emergence of this strain in the UK was associated with travel to Sub-Saharan Africa [30]. A study by Parsons et al. on *Salmonella enterica* serovar Typhimurium ST313 revealed a possibility of pseudogenes degradation linked to higher virulence in the African strain than the strains in advanced settings [31]. Feasey et al. reported two *Salmonella enterica* serovar Enteritidis strains that are restricted to Africa and noted that these strains differed from the S. Enteritidis strains circulating worldwide by possessing diverse virulence determinants and multidrug resistance [23]. 

## 6. Emergence and Causes of Antibiotic Resistance by the Non-Typhoid *Salmonella*

The burden of antibiotic resistance originates in animals but is disseminated by the occurrence of traces of antibiotics in the environment and inappropriate use of antibiotics in human medicine. The use of antibiotics in the agricultural sector has led to the traces of antibiotics being left in soil and water resulting in resistance by the environmental bacterial isolates [32,33]. Soil fertilization using animal fecal matter spreads pathogens and traces of antibiotics in soil and results in contamination of plants and the surrounding environment during watering. *Salmonella enterica* serovar Typhimurium and *Salmonella enterica* serovar Enteritidis are the main non-typhoidal *Salmonella* serovars that have been isolated from the stools of patients [20]. As stated previously, several epidemiology studies have focused on characterizing *Salmonella* isolates from humans, the environment, and animals to trace the origin of isolates that are found in clinical settings [27,29]. Antibiotic resistance genetic determinants that are carried by the *Salmonella* spp. have also been characterized by nucleic acid-based approaches such as polymerase chain reaction and whole-genome sequencing. Similar genetic determinants for resistance to beta-lactams, aminoglycosides, tetracyclines, and fluoroquinolones have been detected in *Salmonella* isolates from livestock and humans [27,34,35]. This led to the conclusion that livestock animals are carriers of antimicrobial-resistant *Salmonella* spp. moreover, food, and environmental contamination are the sources of transmission to humans [34]. 

## 7. Resistance to Current Antibiotics of Last Resort

Following resistance to traditional antibiotics, macrolides, higher generation cephalosporins, and extended spectrum beta-lactams have been the imperative antibiotics against *Salmonella* [36]. The rising trends of multidrug resistance and resistance to fluoroquinolones and 3rd generation cephalosporins in clinical isolates led to the introduction of carbapenems and colistin as critical antibiotics of last resort [36]. 

## 8. Carbapenem Resistance

Beta-lactam antibiotics contain the lactam ring which weakens the bacterial cell wall by disrupting transpeptidase activity during cell wall synthesis [37]. Bacteria developed resistance to beta-lactams by producing beta-lactamase enzymes which degrade the lactam ring through hydrolysis. Extended spectrum antibiotics including cephalosporins, which are resistant to lactamases were then introduced to broaden the activity of beta-lactam antibiotics [38]. However, extended spectrum beta-lactamase enzymes encoded by *blaCMY2*, *blaSHV*, and *blaTEM* emerged thus reducing the efficacy of cephalosporins [37]. Carbapenems are a broader class of extended-spectrum beta-lactams that are resistant to lactamases produced by bacteria [39]. The in vivo activity of carbapenem antibiotics was reported by Capoor et al. and were accepted for treatment of multidrug-resistant Gram-negative pathogens, including *Salmonella* [40]. 

As a result of quick adaptation and evolution, bacteria have developed resistance to carbapenems by producing carbapenemase enzymes that hydrolyze carbapenems [37,41]. Carbapenemase enzymes are classified into two groups based on the amino acid content on their active site i.e., serine beta-lactamases and metallo beta-lactamases [39]. The former contains serine while the later contains zinc ions at their active sites [39]. Additionally, a mutation on bacterial *ompR* enabled Gram-negative bacteria to lose porin channels, which are an entry of carbapenems resulting in less intake of the antibiotic [37,41]. In attempts to preserve their effectiveness, the use of carbapenems in livestock is prohibited worldwide [42]. Carbapenem resistance was first reported in *Klebsiella pneumonia* and *Escherichia coli* clinical isolates and was later described to be plasmid-mediated in Gram-negative bacteria [41,43]. Emergence of resistance towards these antibiotics in human medicine signifies improper use of antibiotics at the clinical settings as pivotal in propagating antimicrobial resistance. 

Surprisingly, carbapenem resistance has also been reported and metallo-beta lactamase genetic determinants such as New Delhi Metallo-β-lactamase (NDM) and Verona integron encoded Metallo-β-lactamase (VIM) have been detected in bacteria isolated from pigs and poultry [44,45,46]. Furthermore, *blaVIM-1* gene was detected in a *Salmonella* isolate from a wild bird in Germany [33]. The *blaVIM-1* and *blaNDM-5* genes were reported to be responsible for resistance to 3rd generation cephalosporins, which are used in animal husbandry; these same genes confer resistance to carbapenems and have been detected in *Salmonella* from food animals [47,48]. Findings from the previous studies support the idea of co-resistance. Carbapenem resistance genes have been recently isolated in different *Salmonella* serovars from different hosts, including food animals as shown in Table 1, signifying their distribution.

These genetic determinants are located in the plasmids, which facilitates their transmission between even unrelated bacterial species (Table 1). Although the clinical setting is the major contributor to carbapenem resistance, the presence of the carbapenem resistance genetic determinants in food animals is an issue of concern since transmission of these genetic determinants to humans is highly feasible. 

## 9. Colistin Resistance

Colistin (polymyxin E) is an antibiotic under the class of polymyxins, which is listed as one of the critically important antibiotics by the WHO [36]. Colistin was first used for veterinary purposes only; however, due to the decreasing efficacy of available drugs in human medicine and the increasing burden of multidrug-resistant bacteria, colistin was recommended as an antibiotic of last resort in human medicine [51]. Colistin disrupts the negative charge of the outer membrane of Gram-negative bacteria resulting in leaking of cytoplasmic contents and cell death [52]. Prescription of colistin for both human and veterinary use has led to the emergence of mobilized colistin resistance (*mcr*) genes, which encode phosphoethanolamine transferase in *Enterobacteriaceae* [51,53]. As a mechanism of resistance Gram-negative bacteria produce phosphoethanolamine transferase, which adds the positively charged phosphoethanolamine to the lipid A of the outer membrane, thus reducing the binding site for colistin [52]. The first report of the *mcr* gene was from China in *Escherichia coli* isolated from pigs [51]. Thus far, 9 variants of *mcr* genes have been detected in *Salmonella* isolated from humans and animals [53,54,55]. 

The *mcr* genes are carried by the conjugative plasmids, which facilitate their transfer between bacterial species [51]. The detection of *mcr* genes in bacteria isolated from food-animals and humans in different countries places colistin at risk of reduced efficacy against Enterobacteria. In South Africa, colsitin *mcr* gene resistance have been detected in bacteria isolated from hospitalized patients, while in countries like Taiwan, China, England, and Germany, variants of *mcr* genes have also been reported in bacteria isolated from pig, cattle, and poultry (Table 2). 

## 10. Genomic Approaches in Monitoring the Dissemination of Antimicrobial Resistance 

Genome-based approaches such as polymerase chain reaction (PCR), pulse-field gel electrophoresis (PFGE), multi-locus sequence typing (MLST), single nucleotide polymorphism (SNP) genotyping, and whole-genome sequencing (WGS) are an accurate way of typing pathogens. These methods enable scientists to draw more clear conclusions on identification and relatedness of AMR pathogens isolated from animals, food, and humans [73]. The high level of discrimination between even related species obtained by genomic approaches generates thorough results which are helpful during outbreak tracing.

PFGE revealed genetic relatedness between *mcr-1* carrying *Salmonella enterica* serovar Typhimurium isolated from humans, pigs, and chicken in Taiwan [59]. WGS was used to reveal high similarity between *mcr-1* carrying plasmid recovered in *S. Typhimurium* isolated from pigs (Great Britain) and a reference *mcr-1* carrying plasmid recovered in *Escherichia coli* isolated from a pig in China [56]. WGS with SNP-based phylogeny analysis uncovered the association between S. Infantis isolated from broiler chickens and *Salmonella enterica* serovar Infantis infecting humans in Italy [58]. PFGE, MLST, and PCR-based plasmid-typing clustered *mcr-1* carrying *Salmonella enterica* serovar Typhimurium ST34 recovered from a duck and pigs from diverse geological sites into one group [61]. From the findings of the authors, there is a relationship between bacteria isolated from animals and humans. Moreover, some studies reported that similar plasmids, such as IncI2, IncX4, and IncHI2 like *mcr* carrying plasmids recovered from *Salmonella enterica* serovar Typhimurium, were detected from humans and food-animals [59,60,61,64]. Genomic methods are valuable tools for tracing pathogens and their associated AMR genes therefore, for proper monitoring and surveillance of *Salmonella*, it is vital to include such methods. 

### 10.1. Pulse-Field Gel Electrophoresis 

PFGE generates DNA fingerprints by using enzyme restriction which recognizes the specific site and digests DNA into fragments of different length. Bacterial strains are then grouped into clones which show similar restriction patterns. This method has been the “gold standard” for typing pathogens due to its proven high level of genetic discrimination of bacteria of the same species. PFGE is used in outbreak tracing and epidemiology studies by the Centers for Disease Control and Prevention [74]. Molecular analysis studies which included subtyping *Salmonella enterica* serovar Poona, *Salmonella enterica* serovar Typhimurium, and *Salmonella enterica* serovar Enteritidis isolated from different sources in diverse geological areas reported PFGE to provide the highest discriminatory power among other genotyping methods tested [46,73,75]. Furthermore, PFGE provided results that were in accordance with WGS in subtyping S. Poona [73]. On the contrary, other studies reported that PFGE was less powerful in subtyping *Salmonella enterica* serovar Heildeberg and *Salmonella enterica* serovar Typhimurium [76,77]. 

### 10.2. Multi-Locus Sequence Typing 

This approach is based on sequencing of housekeeping genes for a bacterial species. The traditional MLST approach is based on PCR amplification, followed by sequencing, and identification of allele variations in seven housekeeping genes for *S. enterica* [78]. Advanced MLST approaches are based on sequencing and identification of core genome loci variations (cgMLST) and pan genome or whole genome (wgMLST) variations in a bacterial species [79]. Whole genome MSLT and cgMLST provide higher resolution than PCR-based MLST as many genes are involved in the former approaches [80]. Furthermore, advanced MLST approaches are more accurate than PCR-based MLST because they are less laborious, resulting in minimum risk of errors. Even though PCR-based MLST is more laborious than the advanced MLST methods, it is a more economic and easily accessibly method in developing settings [80]. PCR-based MLST was used to successfully identify known *S. enterica* isolates with the results correlating to the traditional serotyping method [80].

Comparative studies which revealed the power of MLST over other molecular genotyping approaches in subtyping *Salmonella* include separating outbreak S. Heidelberg isolates into four distinct phylogenetic clusters, which were indistinguishable by PFGE [77]. In another study, MLST was able to predict serovars for 42/46 *Salmonella* isolates, providing better results than PFGE, rep-PCR, and ribotyping [76]. Further genotyping investigations which utilized MLST revealed human and food animal *Salmonella enterica* serovar Typhimurium ST19, *Salmonella enterica* serovar Kentucky ST198, and *Salmonella enterica* serovar Enteritidis ST43 isolates which were indistinguishable, reinforcing circulation of pathogens between food animals and humans [81,82]. *Salmonella enterica* serovar Typhimurium ST19 and ST34 are strains which are associated with MDR, including carbapenem and colistin resistance, and are circulating between humans and food animals [48,49,71,72,82].

### 10.3. Single Nucleotide Polymorphism Genotyping

SNPs are conservative single nucleotide base changes in the genome, which can be synonymous or non-synonymous [83]. The polymorphisms may result in mutations which are responsible for antimicrobial resistance. In pathogen typing, SNPs are used to cluster bacterial strains into clades or haplotypes. PCR-based SNP genotyping detects single base polymorphisms in the targeted loci of interest while whole genome SNP analysis detects polymorphisms throughout the sequenced genome. Comparative studies have shown SNP analysis to be another valuable and powerful approach for typing closely related strains of *Salmonella* [84,85,86,87]. The discriminatory power of PCR-based SNP typing was evidenced in subtyping S. Enteritidis PFGE type XAI.0003 into eight different clades and phage type PT8 into seven clades in *Salmonella* isolates from various sources including food and poultry in Canada [86]. In another study, SNP analysis detected polymorphisms which resulted in different amino acid sequences in the alleles of housekeeping genes in clinical and food *Salmonella enterica* serovar Saintpaul isolates [84]. Although the results were congruent with PFGE, SNP analysis provided further discriminatory power among the clones [84]. In a WGS-based SNP analysis where clinical and pork isolates of S. Derby were subtyped in Germany, this method proved to be of high discriminatory power and an appropriate tool for strains with partial epidemiological data [87]. 

WGS is the most accurate discriminating approach among the genotyping methods however, its worldwide use is limited due to high costs, lack of resources, and expertise in some developing settings [87,88]. Furthermore, WGS requires extensive computing and storage for data generated, adding to the cost of the method [87]. For these reasons, detection of marker sequences by PCR is the most applied approach in research. PFGE and MLST provide more in-depth discrimination between even strains of the same bacterial species. Moreover, approaches such as PFGE, MLST, SNP genotyping can be used to trace and relate pathogens of different geographical areas and hosts. The accuracy of discrimination of molecular genotyping methods differs with bacterial species and strains. There is no one genotyping method that is better than the other. Additionally, one approach can be powerful in discriminating a certain bacterial species and be less accurate in another species. 

## 11. Mobile Genetic Elements Characterization and Antimicrobial Resistance Monitoring

AMR genes are often carried by mobile genetic elements that integrate into plasmids or the bacterial chromosome. Insertion elements and transposons have been found to be responsible for disseminating gene cassettes that confer resistance to antibiotics [56,57]. AMR genes are frequently flanked by the insertion elements or carried along with composite transposons [69,89,90]. These elements have been identified in plasmids that have been isolated from *Salmonella* in humans, food, and animals. The role of IS26 and ISCR1 in mobilizing carbapenem resistance genes was highlighted [50,90]. 

Since plasmids mediate lateral transfer of AMR genes to different hosts and between bacterial species, it is crucial to characterize the plasmids that are carried by pathogens. The plasmids which carry AMR genes are frequently conjugative, as shown by several studies [50,67,71]. Although some plasmids are host restricted, some can be transferred and replicated in different hosts. Similar plasmids have previously been isolated from different hosts and different *Salmonella* serovars, e.g., IncI2 *mcr-1* carrying plasmid, which has been identified from S. Typhimurium and S. Indiana from poultry, pigs, and humans in China [59,90]. A *blaNDM-5* carrying IncX3 plasmid with 99% identity to a *blaNDM-5* carrying plasmid in India was isolated from a pig in China, implying cross-contamination between humans and food animals and broad dissemination of AMR genes by pathogens [48]. 

**Figure 1:**
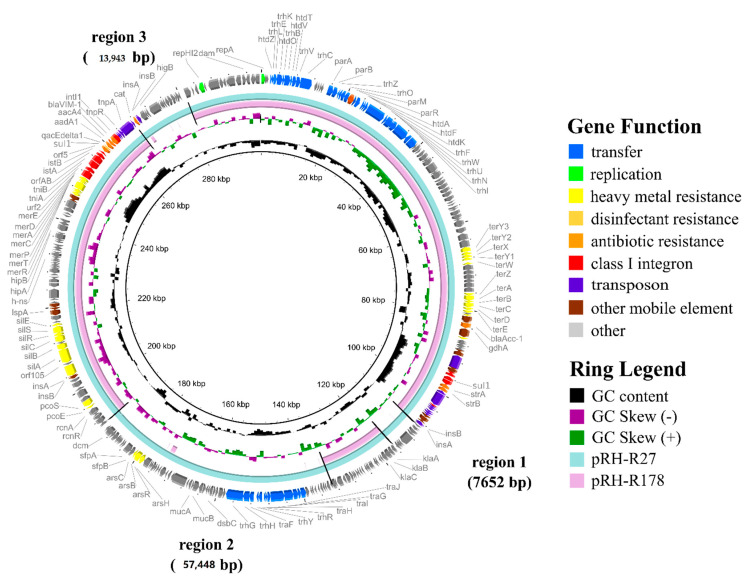
Comparative plasmid maps of pRH-R27 from *Salmonella enterica* and pRH-R178 from *Escherichia coli*. Deleted regions (1–3) are indicated on the map and pRH-R27 was used as reference. All sequences show >95% DNA sequence identity. Coloured regions represent functions and features indicated in the figure legend. The IncHI2 plasmid harbored by S. Infantis (pRH-R27) isolated from a livestock farm in Germany carries antibiotic resistance genes (*blaVIM-1*, *aacA4*, *aadA1*, *sul1*, *blaACC-1*, *strA/strB*, and *catA1*), disinfectant resistance (*qacE*Δ*1*) and heavy metal resistance (*ter-*, *mer-*, *sil-*, *ars-*, *rcn-*, and *pco*). The figure was reproduced with permission from [47].

Plasmid characterization does not only detect the specific genes of interest, but also other antibiotic and heavy metal resistance genes carried by plasmids. For biosecurity, disinfectants are frequently used in meat and poultry production systems. These include quaternary ammonium compounds, heavy metals, phenolic compounds, and alcohols [34]. Due to their moderate toxicity and prominent antimicrobial activity, quaternary ammonium compounds are the commonly used disinfectants [34]. Besides disinfection, heavy metals are also used as growth promoters especially in poultry production. Zinc, copper, organic arsenicals, and silver nanoparticles are added in feed lots and water for animals while mercury is a disinfectant in farms and meat production environments [34,91]. Since high concentrations of heavy metals are toxic to bacteria prolonged exposure to such substances resulted in defense mechanisms by bacteria. Stress response proteins which result in Efflux pumps, heavy metal and disinfectant resistance genes have been identified in livestock-associated bacteria including *Salmonella* [47,92,93,94]. The same mechanisms for resistance to heavy metals could result in cross-resistance to some antibiotic compounds [91]. Genes that encode mercury, silver, arsenate, and cobalt resistance were detected in a *blaVIM-1* carrying InHI2 plasmid isolated from *Salmonella enterica* in pigs as shown in Figure 1 [47]. Detection of resistance by microbiological culturing or PCR alone is not enough to conclude the source of AMR. Further characterization of plasmids coupled with bioinformatics and epidemiology enables determination of the geographical spread of the plasmid.

Plasmid dissemination is mediated by the presence of *tra* genes which encode all the proteins that are needed for the conjugation process [47,89]. Helper plasmids mediate the transfer process in the absence of *tra* genes, such case was reported in the non-conjugative ColE plasmid [95]. Epidemiological studies have shown that *Salmonella* spp. acquire colistin and carbapenem resistance genes mostly from *Escherichia coli* through conjugation [50,67,71,95]. 

## 12. Genotyping of Food Animal Associated AMR *Salmonella* sp. in the Developing Countries

Foodborne diseases affect both developing and developed countries, but the burden is higher in the developing settings. On the other hand, the developing countries primarily lack proper resources for genotyping AMR pathogens or little consideration is given to agriculture-related AMR, leading to paucity of data on the issue. 

In Africa, food animals and farm environments harbor diverse *Salmonella Salmonella enterica* serovars including S. Kentucky, S. Newport, S. Hadar, S. Enteritidis, S. Infantis, and S. Typhimurium [96]. The leading disease causing *Salmonella* serovars S. Typhimurium and S. Enteritidis have been reported in lower prevalence than S. Kentucky, S. Hadar, and S. Newport in food animals in Africa, however, the host species might have an impact on this low prevalence [32,97,98]. Furthermore, like in other continents, extensive distribution of S. Enteritidis and S. Typhimurium has been reported in Africa [96]. According to the cited literature in Africa, poultry, pigs, and cattle are the main reservoirs of the problematic *Salmonella* serovars. Moreover, isolation of multidrug resistant S. Kentucky from food animals is of concern since this serovar has also been isolated from humans [32,97]. Although outbreaks caused by S. Kentucky have not been reported, this serovar is still a threat to humans as it can transfer the AMR genes to other disease-causing pathogens. 

Despite the inaccessibility to proper genotyping resources in Africa, data on genotype profiling of non-typhoid *Salmonella* is accumulating, however, only a few studies have been carried on animal isolates (Table 3). From the genotyping results of *Salmonella*, WGS distinguished outbreak from non-outbreak S. Enteritidis isolates which belonged to common subtypes by PFGE [85]. In another study, subtyping by PFGE clustered isolates S. Newport, S. Mbandaka, and S. Aberdeen from different farms and different districts into pulsotypes PT(H), PT(N), and PT(F), respectively [98]. On the other hand, seven S. Hadar isolates were discriminated into four different pulsotypes and belonged to four farms in two districts [98]. In a study by Fashae et al. MLST showed that humans and cattle share S. Colindale ST584 and S. Kentucky isolates, however, SNP analysis revealed that there was no cluster between human and cattle isolates [97]. MLST detected the global circulating multidrug resistant S. Typhimurium ST19 in isolates from Morocco and PFGE differentiated 9 serovars into 32 pulsotypes, some of which showed up to 5 variations within the same serovar [99].

*Salmonella* has been detected in livestock and poultry from many sub-Saharan African countries including South Africa however, there is still a lack of in-depth knowledge about the multidrug resistant clinically important serovars which are circulating in food chain animals. Data on the predominant serovars are based only on few studies, therefore, more genotyping studies are required in African livestock to understand the epidemiology of *Salmonella* in this continent.

## 13. Discussion

There are rising trends of resistance to carbapenems and colistin in *Salmonella* isolated from food chain animals. Antimicrobial resistant in livestock associated *Salmonella* is an increasing public health concern since carbapenems and colistin are the last-line antibiotics. Of note is that AMR genes which have been isolated from *Salmonella* in animals originated from clinically problematic pathogens such as *K. pneumonia* and *Escherichia coli* [43,51]. Although agriculture, food animals and livestock production systems are significant sources of antimicrobial resistance [5,101,102,103], genetic transfer of AMR through plasmid and mobile genetic elements are the major contributor to the dissemination of AMR genes. More attention should be brought into monitoring the last line antibiotics in animal husbandry, and genomic characterization of the drivers of AMR should be part of the surveillance programs. Antimicrobial resistance is a complex multifactorial challenge requiring a One Health approach of coordinated multidisciplinary efforts to tackle it. It is important to highlight that non-prudent use of antimicrobial agents in human medicine significantly contributes to the development of antimicrobial resistance, underlining the importance of One Health. Active surveillance of foodborne pathogens should be practiced by every country to protect human health and prevent possible outbreaks by emphasizing stringent hygiene measures, especially in food animals. 

Another point of concern is the worldwide dissemination of S. Typhimurium ST34, which carries gene cassettes for MDR, including genes for resistance to carbapenems and Colistin. Particular attention should be given to this serovar as it is found in food animals and responsible for causing diseases in humans. Pigs and poultry are the major reservoirs of carbapenem and colistin resistant *Salmonella*, therefore more stringent surveillance is urgently required in livestock. Although genomic characterization of livestock-associated *Salmonella* has been done in Africa, there is still some lack of information with regards to investigating resistance to the last-line antibiotics and other antibiotics of high clinical importance as listed by the WHO. In addition, disinfectant resistance and heavy metal resistance is an emerging concern in livestock production as it disrupts pathogen control measures. 

Successful surveillance and strict monitoring of colistin and carbapenem resistance in livestock can be achieved by microbiological, genomic analysis, and phylogenetic studies to early detect trends and emergence of resistance which will eventually mitigate antibiotics use in livestock and create *Salmonella* references to be used during disease outbreak tracing. Resistance plasmids offer survival advantages to pathogens by conferring resistance to antibiotics, heavy metals, and disinfectants therefore, plasmid typing in livestock-associated pathogens is important in detecting food related threats to humans. The use of antibiotics in livestock varies with geographical locations therefore different *Salmonella* serovars which carry distinct AMR genes dominate diverse geographical locations. This brings out the need to constantly monitor antimicrobial resistance and genetically characterize AMR pathogens in both livestock and humans. As reported in literature, Africa is leading with foodborne infections, yet it still lacks proper monitoring of foodborne pathogens. Hence, improved surveillance needs to be performed in this continent to reduce transmission of livestock-associated AMR pathogens to humans. 

## Figures and Tables

**Table 1:** *Salmonella* serovars carrying carbapenem resistance genes in food animals and humans.

*Salmonella* Serovar	Host	Plasmid Description	Location	Reference
Infantis	Pig	*blaVIM-1* carrying IncHI2 type	Germany	[44,47]
Typhimurium	Pork	*blaNDM-5* carrying IncX3 type	China	[48,49]
Lomita	Human	*blaNDM-1* carrying IncX3 type	China	[50]
Indiana	Chicken carcass	*blaNDM-1* carrying plasmid	China	[45]
Corvallis	Wild bird	*blaNDM-1* carrying IncA/C type	Germany	[33]

**Table 2:** *Salmonella* serovars carrying plasmid-mediated *mcr* genes from food animals and humans.

*Salmonella* Serovar	Host Species	Plasmid Identified	Location	Reference
Typhimurium	Pig	*mcr-1* carrying like pHNSHP45 (IncI2)	Great Britain	[56]
Typhimurium	Pig and meat	*mcr-5* carrying IncX1 and ColE plasmids	Germany	[57]
Infantis	Broilers and broiler meat	*mcr-1* carrying IncX4	Italy	[58]
Typhimurium	Chicken, pig, humans	*mcr-1* carrying IncI2 type plasmid	Taiwan	[59]
Typhimurium	human	*mcr-1* carrying IncX4, IncI2, and IncHI2	England and Wales	[60]
Typhimurium	Pig, chicken	*mcr-1* carrying IncI2 and IncHI2 type	China	[61]
Schwarzengrund	Poultry meat	*mcr-1* carrying IncX4	Brazil	[62]
Indiana	Poultry	*mcr-1* carrying IncI2 type	China	[63]
Typhimurium	Pig	*mcr-1* carrying IncHI2 like plasmid	China	[64]
Rissen and Typhimurium 1,4,[5],12:i−	Pig	*mcr-1* carrying IncX4 and IncHI2	Portugal	[65]
Typhimurium	Pig	*mcr-4* carrying non-conjugative colE plasmid	Italy	[66]
Typhimurium	human	*mcr-3* carrying IncHI2	Denmark	[67]
Typhimurium	human	*mcr-4* carrying ColE-like plasmid	Italy	[66,68]
Typhimurium and Saintpaul	food	*mcr-1* carrying IncX4	Brazil	[69]
Typhimurium	human	*mcr-1* carrying IncHI2, IncI2 and IncX4 plasmids	China	[70]
Typhimurium	human	*mcr-3* carrying IncHI2	United States	[71]
Typhimurium	Pig	*mcr-1* carrying IncHI2	China	[72]

**Table 3:** Studies which used genotyping approaches to report antimicrobial resistant *Salmonella* serovars in food animals in Africa.

Source	*Salmonella* Serovars Detected	Genotyping Approach	Resistance to Antibiotics or Resistance Genes Detected	Geographic Area	Reference
Poultry	Newport, Heidelberg, Aberdeen, Hadar, Zanzibar, Bolton, Enteritidis, Mbandaka, Typhimurium	PFGE PCR	*blaTEM-1*, *cmlA*, *tetA*, *qnrS*, *sul1*, *dhfrI*, *dhfrVII*	Uganda	[98]
Ruminants, pigs, poultry, environmental and wastewater from farms and humans	Enteritidis, Haifa, Heidelberg, Kentucky, Newport, Senftenberg, Stanleyville, Typhimurium, Virchow	MLVA PFGE	ampicillin; amoxicillin/clavulanic acid; chloramphenicol; kanamycin; streptomycin; sufisoxazole; trimethoprim/sulfamethoxazole; tetracycline; ciprofloxacin; nalidixic acid	Uganda	[32]
Cattle and human	Colindale, Corvalis, Kentucky and other rare serovars	WGS-SNP typing MLST	*blaTEM-1B*, *aac(3)-Id*, *aadA7*, *strA*, *strB* and *tetA*	Nigeria	[97]
Food (including beef, poultry)	Infantis, Mbandaka, Bredeney, Blockley, Typhimurium, Indiana, Hadar, Anatum, Enteritidis, Altona, Senftenberg, Kentucky, Cerro	PFGE MLST and PCR	*aadA2*, *strA*, *sul1*, *sul2*, *floR*, *blaTEM-1*, *blaPSE-1*, *tetA* and *tetG*	Morocco	[99]
Livestock (cow and chicken)	*Salmonella enterica* strains MEZSAL74 and MEZSAL81	WGS, MLST, PCR	Aminoglycosides, fluoroquinolones	South Africa	[100]

## Data Availability

Not applicable
